# An ^1^H NMR- and MS-Based Study of Metabolites Profiling of Garden Snail *Helix aspersa* Mucus

**DOI:** 10.3390/metabo10090360

**Published:** 2020-09-02

**Authors:** Nikolay G. Vassilev, Svetlana D. Simova, Miroslav Dangalov, Lyudmila Velkova, Venceslav Atanasov, Aleksandar Dolashki, Pavlinka Dolashka

**Affiliations:** Institute of Organic Chemistry with Centre of Phytochemistry, Bulgarian Academy of Sciences, 9, Acad. G. Bonchev Str., 1113 Sofia, Bulgaria; sds@orgchm.bas.bg (S.D.S.); m.dangalov@orgchm.bas.bg (M.D.); lvelkova@orgchm.bas.bg (L.V.); vatanassov@orgchm.bas.bg (V.A.); adolashki@orgchm.bas.bg (A.D.)

**Keywords:** mucus, garden snail *Helix aspersa*, ^1^H NMR, metabolites, mass spectrometry

## Abstract

Metabolic profiling based on ^1^H nuclear magnetic resonance (NMR) spectroscopy was applied with the aim to investigate the functional role of the metabolites in lyophilized mucus from the garden snail *Helix aspersa*. Twenty metabolites were unambiguously identified by ^1^H, 1D TOCSY, 2D J-resolved, 2D COSY, and 2D HSQC NMR spectra with water suppression. The metabolic profiles of two fractions with low molecular weight (Mw < 1 kDa and Mw < 3 kDa) are very similar. Metabolites with known antioxidant, antibacterial, and antimicrobial activity were detected by NMR metabolic analysis of mucus samples from *Helix aspersa*. Some of them were confirmed by mass spectrometric analysis. The primary structure of several peptides was identified in low molecular weight fractions (Mw < 1 kDa) by tandem mass spectrometry.

## 1. Introduction

In the last 20 years, numerous research studies have focused on the isolation and characterization of active substances from different natural sources. Snail has proven to be an invaluable source of a number of biologically active components [1,2,3,4]. Some of them are hemocyanins and peptides isolated from the marine snail *Rapana venosa* from the Black Sea and the garden snail *Helix lucorum* [5,6,7,8]. The structure and properties of these substances have been analyzed by different modern methods, and the possibility of their application in different products has been established [9,10,11,12].

Both the mucus and hemolymph of snails are complex mixtures of peptides, glycopeptides, and proteins with diverse biochemical and pharmacological properties [2,3,4]. The hemolymph of Bulgarian garden snails *Helix lucorum* and *Helix aspersa* provides hemocyanins, which perform the same function as hemoglobin, namely, to carry oxygen to all cells in the body. In contrast to other hemocyanins, three isoforms (β-, αN- αD-HaH, and β-, αN- αD-HlH) with a molecular weight (Mw) of about 450 kDa were isolated from both hemocyanins *Helix lucorum* and *Helix aspersa*, respectively [7]. Hemocyanins rom *Helix lucorum* and *Helix aspersa* can be carriers of poorly immunogenic antigens, and are promising adjuvants for viral and bacterial vaccines and their use as novel antitumor, antibacterial, and immunotherapeutic agents has also been established [13,14,15].

Several bioactive compounds as peptides and glycopeptides with antioxidant and antibacterial activity have been identified in the mucus of the garden snail *Cornu aspersum* [2,3,4]. However, the presence and functional role of serum metabolites have been insufficiently studied. So far, only the metabolic profile of the hemolymph from *Mytilus galloprovincialis* has been examined by NMR spectroscopy [16]. Recently, researchers have focused on the study of bioactive compounds from the hemolymph and mucus of marine and garden snails. A number of shellfish studies have been conducted to clarify the physiological roles of hemolymph serum proteins and hemocytes. A comparative ^1^H NMR-based metabolomics was applied to analyze changes in the hemolymph metabolites of resistant silkworm strains after BmNPV inoculation [17]. The hemolymph-specific metabolite profiles in five *Drosophila* species before and after cold exposure were presented by Olsson et al. [18]. Helix complex snail mucus with a proliferative effect has also been presented [19,20].

Recently the ^1^H NMR spectra showed characteristic metabolic profiles of isolated organs (kidney, heart, digestive gland, and pulmonary membrane) in the snail *Helix aspersa maxima* [21]. The low molecular weight metabolites of metabolic pathways were affected by toxic compounds.

In the present study, we focused on the profiling of low molecular weight fractions from the mucus of *Helix aspersa* (Mw < 1 kDa and Mw < 3 kDa), which has not been studied so far. This is the first report of low molecular weight metabolites identified in the mucus of garden snails. Knowledge of the active ingredients and their action in the mucus of *Helix aspersa* is valuable for their potential application in cosmetology and/or pharmacy.

## 2. Results

The crude mucus extract was collected from the garden snail *Helix aspersa* and two fractions were obtained by ultrafiltration on membranes 3 and 1 kDa. The fractions with a molecular mass below 3 kDa and below 1 kDa, respectively, were purified and analyzed. After lyophilization and concentration, the obtained fractions were analyzed by mass spectrometry and NMR to determine the primary and secondary metabolites.

### 2.1. ^1^H-NMR of Mucus from Helix aspersa

**Figure 1:**
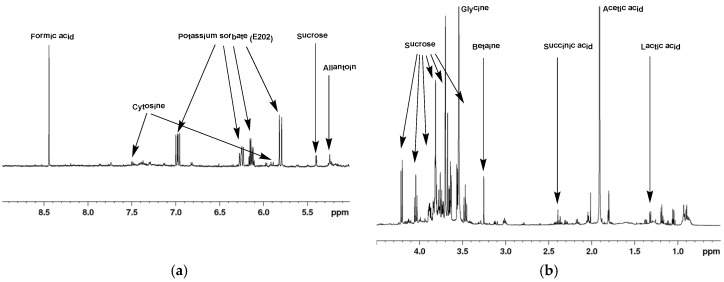
Typical ^1^H-NMR spectrum of lyophilized mucus from *Helix aspersa* (<1 kDa) with assignment of the main metabolites. (1D-noesy experiment with water presaturation, pH = 7.35, 298.0 K). (**a**) ^1^H-NMR spectrum in the interval from 5.0 to 9.0 ppm; (**b**) ^1^H-NMR spectrum in the interval from 0.5 to 4.5 ppm.

**Figure 2:**
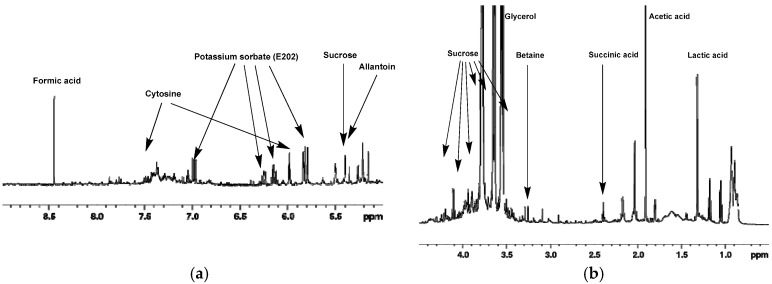
Typical ^1^H-NMR spectrum of lyophilized mucus from *Helix aspersa* (<3 kDa) with assignment of the main metabolites. (1D-noesy experiment with water presaturation, pH = 7.35, 298.0 K). (**a**) ^1^H-NMR spectrum in the interval from 5.0 to 9.0 ppm; (**b**) ^1^H-NMR spectrum in the interval from 0.5 to 4.5 ppm.

Typical ^1^H-NMR spectra of mucus from *Helix aspersa* (<1 kDa and <3 kDa) are shown in Figure 1 and Figure 2. The spectra contain sharp signals for the metabolites with low molecular weight in the aliphatic region between 4.5 and 0.5 ppm; however, in the aromatic region, no signals or only such with low intensity are shown. In general, the 1D NMR spectra show very good reproducibility of the chemical shifts due to the maintenance of a uniform pH by adding phosphate buffer to the sample. Preliminary experiments were made to evaluate the metabolic stability. Freshly prepared samples of lyophilized mucus from *Helix aspersa* (<3 kDa) were acquired immediately and after 24 h (sample kept at 298 K). The recorded 1D NMR spectra match very well without any visible changes (Figure S4) and without indications of ongoing biochemical processes.

Unambiguous identification of the signals of some metabolites was achieved using HSQC spectra (Figure S5 and Table 1). Analysis of the COSY spectra (Figure S6) confirmed the presence of these metabolites. The inspection and database matching of 1D-spectra identified nine additional metabolites. Fourteen additional metabolites were unambiguously identified by 1D selective TOCSY experiments with water suppression. Table 1 does not include the signals that were unassigned or ambiguously assigned. Previous studies of hemolymph from *Mytilus galloprovincialis* indicate that osmolytes (betaine and glycine) and nutrients (glucose and amino acids, such as alanine, valine, and others) were the metabolites with high concentrations in mucus [16].

### 2.2. Mass Spectrometric Analysis

**Figure 3:**
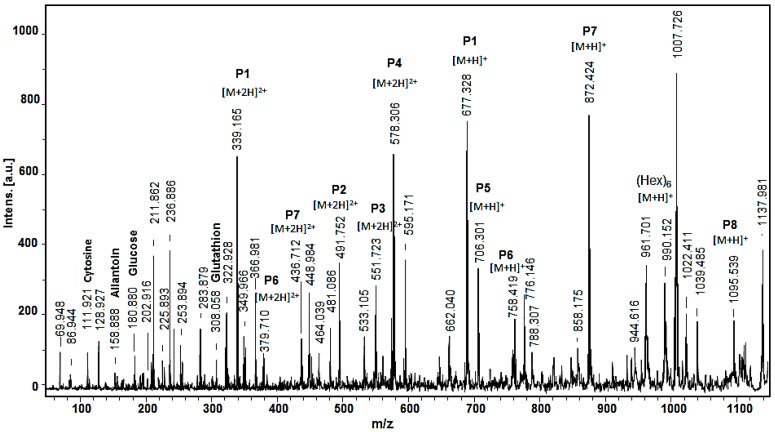
MALDI-MS spectrum of the fraction with compounds with Mw < 1 kDa from the mucus of the garden snail *Helix aspersa*. Standard peptide solution was used to calibrate the mass scale of the AutoflexTM III, High Performance MALDI-TOF & TOF/TOF Systems (Bruker Daltonics, Bremen, Germany).

Matrix-assisted laser desorption/ionization time-of-flight mass spectrometry (MALDI-TOF/MS) was used to determine the molecular mass of compounds in the mucus fraction below 1 kDa. The MALDI-MS spectra (Figure 3) confirmed the presence of the amino acids serine at *m*/*z* 86.94, cytosine at *m*/*z* 128.93, allantoin at *m*/*z* 158.89, and glucose *m*/*z* 180.88, identified by ^1^H-NMR analysis. Glucose and glycogen were previously also detected at low concentrations in hemolymph from *Mytilus galloprovincialis* [16]. It is known that approximately 8–12 glucose units linked linearly by α(1→4) glycosidic bonds form the linear chains of a branched biopolymer glycogen [23]. Glycogen can be broken down to trehalose as an energy source for tissues, which is secreted into the hemolymph. On the other hand, two mucus-producing gland types have been described in the ventral region (foot sole) of *Helix aspersa* and *Helix pomatia*, which react positively for sugars, such as mannose and fucose [24]. Therefore, the single charged ion [M+H]^+^ at *m*/*z* 990.150 in the MALDI-MS spectrum (Figure 3) could correspond to six hexoses linked together linearly in the chain length according to the previous data.

Furthermore, the presence of glutathione (GSH) was detected at *m*/*z* 308.058 Da [M+H]^+^ by MALDI-MS analysis, which is a tripeptide of cysteine, glutamate, and glycine with a gamma peptide linkage between the carboxyl group of the glutamate side chain and cysteine. The sulfhydryl group (−SH) of the cysteine is involved in reduction and conjugation reactions for the removal of peroxides and many xenobiotic compounds. GSH is the major endogenous intracellular antioxidant [25], which recently was also found in hemolymph serum of mussels *Mytilus galloprovincialis* [15].

As shown in Figure 3, the MALDI-MS spectrum of the mucus from *Helix aspersa* is dominated by peptides, shown primarily as doubly charged ions [M+2H]^2+^ at *m*/*z* 339.165, *m*/*z* 578.305, and *m*/*z* 872.424 and single charged ions [M+H]^+^ at *m*/*z* 706.301 and at *m*/*z* 1098.53.

**Figure 4:**
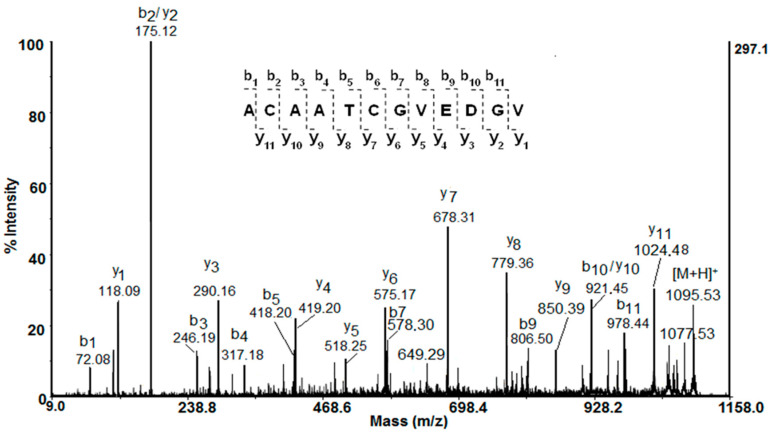
MALDI-MS/MS spectrum of fragmented peptide at *m*/*z* 1095.53 Da (positive ion mode).

The amino acid sequences of the peptides were identified by de novo MALDI-TOF-MS/MS sequencing experiments of the protonated molecule ions. The primary structure of the peptide detected as a double church ion [M+H]^+^ at *m*/*z* 1095.539 Da is shown in Figure 4. Following the series of y- and b-ions from the MALDI-TOF/MS/MS spectrum, the sequence ACAATCGVEDGV was deduced (Figure 4). The primary structures of eight peptides from the fraction below 1 kDa, shown in Table 2, were identified by their MALDI-MS/MS spectra. The obtained structure of the peptide P6 at *m*/*z* 379,710 [M+2H]^2+^ fully corresponds to the structure previously defined by an LTQ Orbitrap XL mass spectrometer [4]. Peptide sequencing performed by MS/MS spectra showed that peptides rich in glycine (G), leucine (L), alanine (A), asparagine (N), valine (V), and proline (P) are present in the mucus (Table 2). The isoelectric points (pI) and monoisotopic molecular mass of the peptides were predicted by the ExPASy MW/pI tool program (https://web.expasy.org/compute_pi/) (Table 2).

## 3. Discussion

Snails secrete biological secretion (mucus), which is a rich source of various bioactive natural compounds. The mucus performs various functions in the life of snails—from adhesive, emollient, moisturizing, and lubricating, to protective and regenerating. The antimicrobial activity of the snail mucus of *Helix aspersa* had a strong antibacterial effect against several strains of *Pseudomonas aeruginosa* and a weak effect against *Staphylococcus aureus* [1]. Moreover, the wound healing activity of *Helix* snail mucus exhibits prosurvival, proliferative, and promigration effects on mammalian fibroblasts [19]. In addition, helix complex was able to protect against O_3_ exposure by preventing oxidative damage and the consequent proinflammatory response in both 2D and 3D models [20].

### 3.1. Metabolic Pathway and Activity

The detected metabolites in Table 1 are known to take part in various metabolic pathways in humans, mammals, and plants. The available literature data will be discussed here in conjunction with the detected concentrations of these metabolites in mucus from *Helix aspersa*.

In the ^1^H-NMR metabolic profile of mucus from *Helix aspersa*, the concentration of acetic acid was the highest. This fact is not a surprise because it is known that it is produced by Gram-negative bacilli and Gram-positive cocci [26]. Acetic acid is also found in *Akkermansia*, *Bacteroidetes*, *Bifidobacterium*, *Prevotella*, and *Ruminococcus* [26,27]. The concentration of other microbial metabolites, such as formic acid, lactic acid, and succinic acid, is moderate. Like acetic acid, they are produced by Gram-negative bacilli and Gram-positive cocci [26]. Lactate as a metabolite has been demonstrated in tissue extracts of many invertebrate species, including *A. subfuscus* and *Helix aspersa* [21,28], but for the first time, it was found in the mucus. In mollusks, lactic acid is a minor product of anaerobic glycolysis; in contrast, succinic acid is a major end product. Lactate is an important end product in terrestrial and freshwater gastropods but not in marine species. The biologically active derivatives of unsaturated fatty acids have been detected previously in a large number of invertebrate species, including marine mollusks [29].

In the ^1^H NMR metabolic profile of mucus from *Helix aspersa*, the concentrations of the following metabolites are moderate: 3-hydroxybutyrate (β-hydroxybutyrate) (a product of the normal metabolism of fatty acid oxidation and a convenient carrier of energy from adipocytes to peripheral tissues during fasting or exercise [30]) and 4-methyl-2-oxovaleric acid (alpha-ketoisocaproic acid) (it and its conjugate base, α-ketoisocaproate, are metabolic intermediates in the metabolic pathway for L-leucine [31]).

The concentrations of allantoin (a product of the oxidation of uric acid), betaine, and cytosine are also moderate. The detected allantoin is a major metabolic intermediate in most organisms, including animals, plants, and bacteria. It is a product of purine metabolism and it is produced from uric acid (a degradation product of nucleic acids) by the action of urate oxidase [32]. Allantoin has long been used in cosmetics and in medicine without showing toxicity or undesired side-effects [33]. It is known for its desquamating action, and its promotion of cell proliferation and wound healing [34]. In recent years, research on the secretions of the snail *Helix aspersa* has confirmed that the mucus contains an unusual combination of natural compounds with beneficial and therapeutic qualities for human skin, such as allantoin and glycolic acid [33]. The detected betaine is known to function as a methyl donor and by facilitating the necessary chemical processes. Betaine comes either from food or from the oxidation of choline. Betaine deficiency is associated with metabolic syndrome, lipid disorders, and diabetes, and could have a role in vascular and other diseases [35]. Cytosine as a pyrimidine base is a major building block of nucleic acids. Cytosine is unstable and could change to uracil (spontaneous deamination). It is no coincidence that the nucleotide of cytosine is the prime mutagenic nucleotide in leukemia and cancer [36].

In the tested mucus fraction, only four amino acids (alanine, glycine, phenylalanine, and valine) were found at relatively low concentrations. Alanine is produced in the body from either the conversion of the carbohydrate pyruvate or the breakdown of DNA and the dipeptides carnosine and anserine. It is highly concentrated in muscle and is one of the most important amino acids released by muscle, functioning as a major energy source. It also participates in the metabolism of sugars and organic acids and improves the immune system through its contribution to the formation of antibodies. Glycine strengthens the immune system and controls the release of oxygen during cell formation. This is a fast neurotransmitter inhibitory. Glycine takes part in the production of DNA, phospholipids, and collagen, and in the release of energy in the body [37]. The detected valine along with leucine and isoleucine are essential amino acids. Therefore, valine must be ingested, usually as a component of proteins [36].

Recently, various amino acids, such as glycine, valine, lysine, threonine, asparagine, tyrosine, and histidine, were detected as metabolomics of hemolymph serum of *Mytilus galloprovincialis* by ^1^H-NMR. An increasing presence of Cu^2+^ led to increased levels of glucose and amino acids, including lysine, threonine, serine, glutamine, and alanine [16]. A number of free amino acids (for example, Ala, Arg, Glu, Gly, Leu, and Ile), including taurine, have been found previously in various marine mollusks and gastropods [38]. Free taurine, betaine, and glycine appear to be almost ubiquitous constituents of marine invertebrates.

The detected choline is a basic metabolic component found in many plants and animal organs. It plays an important role as a methyl group donor in various vital metabolic processes but also as a precursor for the neurotransmitter acetylcholine, as well as in lipid transport and metabolism [36]. The concentrations of the following metabolites are also moderate: ethanol, glucose, and sucrose. Ethanol is also detected as a metabolite of other invertebrates *Lumbricus rubellus* (Hoffmeister) and *Eisenia andrei* (Savigny) [28] as well as hemolymph serum from the marine mollusk *M. galloprovincialis* [16]. Ethanol is metabolized by the body as a carbohydrate nutrient that provides energy because it metabolizes into acetyl CoA, an intermediate in glucose metabolism, that can be used for energy in the citric acid cycle or for biosynthesis [36]. The identified glucose belongs to endogenous metabolites and is a major source of energy for living organisms. In animals, glucose arises from the breakdown of glycogen in a process known as glycogenolysis. Sucrose is a nonreducing disaccharide composed of glucose and fructose linked via their anomeric carbons. BioTransformer predicts that sucrose is a product of the metabolism of 6-O-sinapoyl sucrose by a hydrolysis reaction of a carboxylic acid ester occurring in human gut microbiota and catalyzed by the liver carboxylesterase 1 (P23141) enzyme [39].

Classical metabolic vertebrate pathways, such as glycolysis, Krebs cycle, and respiratory chain, have also been described in mollusks [40]. In living organisms, succinic acid is known to be in the form of an anion, succinate, which has multiple biological roles as a metabolic intermediate. Succinate is known as an intermediate of the Krebs cycle, a crucial pathway in energy metabolism described in vertebrates but also in mollusks [40]. The increase of this metabolite could be due to a decrease in Krebs cycle activity. The cases of succinate accumulation could be membrane damage and disruption of the mitochondrial electron transport chain, which endangers the energy production from aerobic respiration [21]. Succinate is also the final product of glucose-succinate and asparate-succinate pathways, which takes over the anaerobic metabolism in invertebrates and can be related to the lactate pathway being described in mollusk’s, such as the blue mussel *Mytilus edulis* [41].

The ^1^H NMR-detected concentration of glycerol in mucus from *Helix aspersa* is high and was detected only in the lyophilized sample (<3 kDa). Glycerol and fatty acids are the basis of the structure of fats. Glycerol can be transformed into glucose, thus providing energy for cellular metabolism [36].

#### 3.1.1. Antibacterial Metabolic Activity of Acetic, Citric, Lactic, Tartaric, and Isovaleric Acids, and Glycerol

Recently the inhibitory effect of four organic acids (acetic acid, citric acid, lactic acid, and tartaric acid) was evaluated at different levels of contamination by *S. typhimurium* [42].

Lactic acid is widely used to inhibit the growth of important microbial pathogens. The antibacterial mechanism of lactic acid was recently studied on *Salmonella enteritidis*, *E. coli*, and *Listeria monocytogenes* by size measurement, TEM, and SDS-PAGE analysis [43]. The results indicated that 0.5% lactic acid could completely inhibit the growth of *S. enteritidis*, *E. coli*, and *L. monocytogenes* cells. Meanwhile, lactic acid resulted in leakage of the proteins of *Salmonella, E. coli*, and *Listeria cells*. The results suggested that the antimicrobial effect of lactic acid was probably caused by physiological and morphological changes in bacterial cells.

A recent study proved the suitability of acetic acid, in a low concentration of 3%, as a local antiseptic agent, especially for use in salvage procedures in problematic infections caused by organisms, such as *Proteus vulgaris*, *Acinetobacter baumannii*, or *Pseudomonas aeruginosa*. It was concluded that acetic acid at a concentration of 3% has an excellent bactericidal effect and, therefore, seems to be suitable as a local antiseptic agent [44]. The antimicrobial effect of pyruvic or succinic acid with oregano essential oil on *Salmonella* survival was assessed [45]. The obtained results indicated that broiler meat treatment with pyruvic or succinic acids with 0.5% essential oil will provide an effective reduction of *Salmonella* and improve raw ground meat quality.

Minimal inhibitory concentrations and antimicrobial effects of glycerol monolaurate (monolaurin), ethanol, and lactic acid, either alone or in combination, against *L. monocytogenes* in tryptic soy broth were recently determined. The data indicated little interaction between the components, when applied in combination against *L. monocytogenes*. The MIC value of lactic acid alone was 5000 μg/mL (0.5%) [46].

#### 3.1.2. Antimicrobial Activity of Peptides

Peptides are important bioactive natural compounds, which are present in the hemolymph and mucus of many mollusk species [2,3,4,7,47]. Some of the identified peptide sequences showed similarity to known antimicrobial peptides (AMPs), which are important components of the innate immune system and act as a first line of defense, providing an immediate response against the large set of various pathogens. Using tandem mass spectrometry, the primary structure of eight peptides in the mucus fraction below 1 kDa was identified (Table 2). Most of the identified peptides contain high level of leucine, glycine, phenylalanine, aspartic acid, asparagine, and histidine as well as one or two proline residues (P3 and P6) inserted into the sequences, which are characteristic to AMPs. The analyses showed that identified peptides from mucus (fraction < 1 kDa) are amphipathic molecules with pI < 7.0. More of them have a neutral net charge and contain between 6 and 15 amino acid residues. Hydrophobic amino acids, such as Trp, Val, Leu, and Ileu, in the peptide sequence are important for the formation of the secondary structure and interactions with the bacterial membrane. Many AMPs are unstructured in free solution and fold into their final conformation upon partitioning with bacterial membranes. Usually, AMPs interact and disrupt the cytoplasmic membrane, but they have also been reported for AMPs, which can pass through the membrane and target the ribosome, internal proteins, or nucleic acids. Recently, novel peptides with molecular masses up to 3 kDa were found in the fraction with antibacterial activity, isolated from the garden snail *C. aspersum* (also known as *Helix aspersa*) [1,3,47], as well as peptides with antioxidant activity [4]. After alignment of the amino acid sequences of peptides shown in Table 2 with database AMPs by CAMPSing (http://www.campsign.bicnirrh.res.in/blast.php), similarity with known nature antimicrobial peptides was found (Information S8 A). Peptide P4 is rich in Gly and Leu residues and shows identities with different fragments of ctenidin-1 and ctenidin-3 (isolated from *Cupiennius salei*; UniProt P86798 and UniProt P86797, respectively), and possessed a defense response to Gram-negative and Gram-positive bacteria. Peptide P7 is rich in Gly and Ala residues and shows identities of 80% with the fragment of AMP cecropin TY1 (from the horsefly *Tabanus yao*; UniProt C1IBY1) (Information S8 A). Moreover, the alignment for peptide 4 in BLAST shows an 86% and 85% similarity with a glycine-rich cell wall structural protein from *Arabidopsis thaliana* and putative protein Ll1785 from snail *Littorina littorea*, respectively. The alignment for peptide 7 in BLAST shows a 92% similarity with the fragment of voltage-dependent calcium channel gamma-8 subunit, partial from *Homo sapiens*. The presented hits demonstrate e-values with intermediate values between 1e-3 and 1 (Information S8 A, B); however, all identified peptide sequences are highly repetitive, which is known to cause overestimates of the e-values in blast alignment.

#### 3.1.3. Antifungal Metabolic Activity of Alpha-Ketoisocaproic Acid, Betaine Derivatives, and Choline

α-ketoisocaproic acid and its conjugate base, α-ketoisocaproate, are metabolic intermediates in the metabolic pathway for L-leucine [31]. The amino acid derivative 2-hydroxyisocaproic acid is a nutritional additive used to increase muscle mass. It has broad antibacterial activity, and recently, a broad antifungal activity against *Candida* and *Aspergillus* at concentrations relevant for topical therapy was reported. As a fungicidal agent with broad-spectrum bactericidal activity, it could be useful in the topical treatment of multispecies superficial infections.

The detected betaine and choline are important for maintaining the osmotic balance in cells. In a recent study [21], an increase of betaine relative to the controls revealed a defense mechanism against oxidative stress and represented a response to the toxic exposure of *Helix aspersa* snails. Choline is one component of phospholipids and, as such, it is important for the structural integrity of cell membranes. Its increased level could be associated with membrane damage induced by reactive oxygen species and free radicals generated by lipid peroxidation [48,49]. Several studies have also suggested that betaine derivatives can be used as antimicrobial agents. A recent study demonstrated the antifungal activity and mechanism of action of a betaine derivative. Lauryl betaine influences ergosterol synthesis in *C. neoformans* and the compound exerts a similar mechanism of action on *M. restricta*.

The antibacterial attributes of a set of choline and geranate (CAGE)-based ionic liquids were reported and the mechanism by which they interact with the Gram-negative cell wall of *Escherichia coli* was identified [50]. The study provides the fundamental mechanism of the action of choline-based ionic liquids on Gram-negative bacteria and demonstrates the promise of CAGE as a powerful antimicrobial agent to treat infections.

### 3.2. Antioxidant Metabolic Activity

The main function of the identified GSH in mucus below 1kDa is to protect cell membranes from oxidative damage by reducing reactive oxygen species and free radicals, i.e., removing them. It is able to prevent damage to important cellular components caused by free radicals, peroxides, lipid peroxides, and heavy metals [24]. Glutathione is an antioxidant in many biological species (plants, animals, fungi, and some bacteria and archaea). The GSH and its related compounds can be used in therapy for a variety of diseases, including viral infection, cystic fibrosis, and cancer [51,52].

The pro- or antioxidant effect of lactate ion was tested recently [53] in vitro at different concentrations. The results suggested that lactate ion could prevent lipid peroxidation by neutralizing free radicals, such as O_2_^−^· and ·OH, but not lipid radicals. Therefore, lactate ion might be considered as a potential antioxidant agent.

Krebs cycle intermediates (KCIs) are reported to act as energy substrates in mitochondria and to manifest antioxidant effects on the brain. A recent study identified that KCIs are effective neuroprotective compounds against oxidative stress in neuronal cells [54]. It was found that pyruvate, oxaloacetate, and α-ketoglutarate, but not lactate, citrate, iso-citrate, succinate, fumarate, or malate, preserve HT22 cells from hydrogen peroxide-mediated toxicity. Because these intermediates did not have any toxic effects (at least up to 10 mM), they can be used in therapeutic treatment of chronic neurodegenerative diseases.

## 4. Materials and Methods

### 4.1. Mucus Collection and Preparation of Different Fractions

The mucus was collected from the foot of *Helix aspersa* snails, grown in Bulgarian eco-farms using patented technology without any snail suffering [55]. A special device was used where the snails were placed with a small amount of distillated water and electrical stimulation with low voltage was applied. This stimulated the snails to release mucus and kept them alive. Using this method, the snails remained alive without disturbing their biological functions and were returned to the farm. The method can be used repeatedly to extract mucus. Thus, the obtained crude mucus extract was homogenized and subjected to centrifugation to remove coarse impurities. The supernatant was subjected to several cycles of filtration, using filters with smaller pore sizes for each subsequent filtration. After several homogenization and purification steps, the subject of an own patent, including filtration and centrifugation for removal of rough particles, the crude mucus extract was separated in two fractions containing bio compound with an Mw below 10 kDa and above 10 kDa. The fraction with an Mw below 10 kDa was the subject of additional separation by ultrafiltration on membranes for 1 and 3 kDa (Millipore™ Ultrafiltration Membrane Filters, Regenerated cellulose). The isolated two fractions from the mucus of the snail *Helix aspersa* containing compounds with a molecule mass below 1 kDa and below 3 kDa were concentrated by lyophilization and analyzed by mass spectrometry and NMR to determinate their metabolic profile.

### 4.2. Analysis by Mass Spectrometry

The two fractions from the mucus of the snail *Helix aspersa* containing compounds with molecule masses below 1 kDa and below 3 kDa were analyzed by an LTQ Orbitrap XL hybrid mass spectrometer (Thermo Fisher Scientific) and the amino acid sequences of a peptide with Mw 1095.53 Da were confirmed by MALDI-TOF-TOF mass spectrometry on an AutoflexTM III. High Performance MALDI-TOF&TOF/TOF System (Bruker Daltonics, Bremen, Germany) uses a 200 Hz frequency-tripled Nd–YAG laser operating at a wavelength of 355 nm. A total of 3500 shots were acquired in the MS mode and a collision energy of 4200 was applied. The mixture of angiotensin I, Glu-1-fibrinopeptide B, ACTH (1–17), and ACTH was used for calibration of the mass spectrometer. The MS/MS spectra were carried out in reflector mode with external calibration using fragments of Glu-fibrino-peptide B. Each sample was prepared by mixing of 2.0 μL of the fraction with 2.0 μL of matrix solution (10 mg/mL of α-cyano-4-hydroxycinnamic acid (CHCA) in 50% ACN containing 0.1% TFA). Then, 1.0 μL of the mixture was spotted on a stainless-steel 192-well target plate. The samples were allowed to dry at room temperature and analyzed. The amino acid sequences of the peptides were identified by precursor ion fragmentation using MALDI-MS/MS analysis.

### 4.3. ^1^H-NMR Spectroscopy

Each sample, consisting of typically 50 mg of lyophilized mucus from *Helix aspersa* (Mw< 1 kDa or Mw< 3 kDa), was extracted using 1 mL of phosphate buffer (pH was adjusted to 7.35 ± 0.05) under stirring for 2 h. The solution was centrifuged for 10 min and 540 μL of natant was transferred into a 5-mm NMR tube and 60 μL of D_2_O containing 1.86 mM 3-(trimethylsilyl)-propionic-d4 acid sodium salt (TSP) was added to yield a final TSP concentration of 0.186 ± 0.05 mM. The sample was kept at 298 K for at least 15 min prior to data acquisition. ^1^H NMR spectra were acquired on a Bruker Avance II+ spectrometer operating at 14.1 T (corresponding to a proton Larmor frequency of 600 MHz), equipped with a Z-gradient 5-mm BBO probe. The temperature was set to 298.0 K, and controlled within ±0.1 K by means of the B-VT 3000 VTU system. All NMR spectra were acquired with water suppression by on-resonance pre-irradiation of the water signal using the 150 Hz bandwidth of the water suppression pulse. The carrier frequency SFO1 was adjusted sample by sample within 0.15 Hz precision for optimal water suppression. The ^1^H NMR spectra were acquired using a 1D-noesy pulse sequence. Typical acquisition parameters included a 5 s relaxation delay, 256 scans, 4 dummy scans, 9615 Hz (16 ppm) spectral width, 64-K complex points, 3.41 s acquisition time, 10 ms mixing time, and a total acquisition time of about 30 min. Data were multiplied by an exponential decay function with a line-broadening factor of 0.3 Hz, prior to Fourier transformation (FT) and manual phase correction. Several experiments were acquired for unambiguous assignment of the metabolites: 1D sequence with presaturation using a composite pulse and spoil gradient [56] in order to achieve better solvent suppression, 1D sequence with water suppression using excitation sculpting with gradients using perfect echo [57,58] in order to get superior suppression of the water signal and to increase the sensitivity, 1D selective TOCSY, 2D J-resolved (JRES), 2D COSY, and 2D HSQC.

Homonuclear 2D-COSY and heteronuclear 2D ^1^H,^13^C HSQC experiments were measured as well on most of the samples to assign the metabolite resonances. Two-dimensional ^1^H,^13^C HSQC NMR experiments were acquired using the hsqcetgpprsisp.3 Bruker pulse sequence (HSQC with sensitivity improvement, echo/anti-echo-TPPI gradient selection, decoupling during acquisition, and presaturation during the relaxation delay). Typical acquisition parameters included: 3.0-s relaxation delay, 64 scans, 16 dummy scans, 0.085 s acquisition time, 145 Hz for direct XH coupling constant, 1024 × 200 complex data points, and 6009 Hz (10 ppm) and 26,410 Hz (175 ppm) spectral widths in F2 and F1. Data were zero-filled to a 1024 × 1024 data matrix and treated with the squared cosine window function (along F2 and F1) prior to FT in phase-sensitive mode. The 2D COSY NMR spectra were acquired using the cosygpmfqf Bruker pulse program (gradient selected, double quantum filter, magnitude mode, adapted with presaturation during the relaxation delay). Acquisition parameters included: 3 s relaxation delay, 16 scans, 16 dummy scans, 0.322 s acquisition time, 4096 × 256 data points, and 6356 Hz (10 ppm) spectral width (in F2 and F1). Data were treated with the sine window function (along both F2 and F1) prior to FT.

One-dimensional selective TOCSY experiments with water suppression were measured as well on most of the samples to identify the metabolites and unambiguously assign the resonances. One-dimensional selective TOCSY experiments were acquired using the seldigpzs Bruker pulse sequence (1D homonuclear Hartman-Hahn transfer using the DIPSI2 sequence for mixing using selective refocusing with a shaped pulse with zero quantum suppression with presaturation during the relaxation delay). Typical acquisition parameters included a 2 s relaxation delay, 1024 scans, 4 dummy scans, 9615 Hz (16 ppm) spectral width, 64K complex points, 3.41 s acquisition time, 80 ms 180 degree Gaussian refocusing pulse, and a total acquisition time of about 30 min.

In addition to the NMR spectra of the lyophilized mucus from *Helix aspersa* (Mw < 1 kDa or Mw < 3 kDa), a blank sample (i.e., buffer only) was measured every time a new stock solution of phosphate buffer was prepared in order to control the levels of contaminants. The typical ^1^H NMR spectrum of a blank sample (negative control) is presented in Figure S7.

### 4.4. Assignment of Metabolites and Database Search

All NMR spectra were processed with Bruker TOPSPIN 3.5 program and preliminary analyzed with the Bayesil web system [59], which is designed for automatic identification and quantification of metabolites using 1D ^1^H NMR spectra of ultra-filtered plasma, serum, or cerebrospinal fluid. The assignment of the metabolite resonances was then manually checked and refined by comparison between experimental 1D NMR spectra and spectra of single metabolites downloaded from “BioMagResBank” [60]. JRES spectra with water suppression were very useful in the assignment of resonances in the case of overlapped multiples (Figures S1 and S2). The 1D selective TOCSY experiments were used for the identification of metabolites with close resonances or in the case of overlap of several multiplets (Figure S3).

## 5. Conclusions

A protocol for the determination of metabolites in mucus from *Helix aspersa* by NMR spectroscopy was developed. The determined metabolic profiles in the two low molecular weight fractions (<1 kDa and <3 kDa) are similar. The compounds identified in two low molecular fractions from *Helix aspersa* mucus include free primary amino acids; sugars, such as glucose and sucrose; intermediates, such as fumarate; osmolites, such as betanine and choline (organic base); and several other organic acids, including isovaleric acid, lactic, tartaric, and acetic acid; as well as alantoin, glutathione, and antimicrobial peptides. Many of these compounds are probably obtained from the snail’s diet while others are biotransformed or de novo biosynthesized by the snails. The type and concentration of metabolites are very similar. Metabolites with known antioxidant, antibacterial, and antimicrobial activity were detected by NMR metabolic analysis and mass spectrometric analysis of mucus samples from *Helix aspersa*. These active metabolites open new horizons for the application of mucus extracts from *Helix aspersa* in cosmetology and/or pharmacy.

## Figures and Tables

**Table 1:** List of the metabolites identified by NMR in lyophilized mucus from *Helix aspersa*, reported as the average from 3 controls.

Metabolite	Mass	^13^C Chemical Shift ^a,b^	^1^H Chemical Shift (Multiplicity, Coupling Constants) ^a,c^	Concentrations in (<1 kDa) Sample (mM)	Concentrations in (<3 kDa) Sample (mM)
3-Hydroxybutyrate	103.1		1.19(d, J = 6.2), 2.29(dd, J = 6.4, 14.3), 2.40(dd, J = 7.3, 14.3), 4.14(sextet, J = 6.4)	0.32	0.06
4-Methyl-2-oxovaleric Acid	130.1		0.93(d, J = 6.5), 2.08(m), 2.60(d, J = 7.0)	0.41	0.49
Acetic acid	60.052	23.45	1.92(s)	16.14	3.51
Alanine	89.09		1.47(d, J = 7.3), 3.77(q, J = 7.3)		0.06
Allantoin	158.121		5.38(s)	0.09	0.04
Betaine	117.148	53.31	3.27(s)	0.15	0.03
Choline	104.1708		3.23(s)	0.02	0.01
Cytosine	111.10		7.51(d, J = 7.4), 5.98(d, J = 7.4)	0.08	0.05
Ethanol	46.07	62.80	3.67(q, J = 7.1), 1.19(t, J = 7.1)	0.33	0.30
Formic acid/formate	46.03		8.46(s)	0.30	0.15
Glucose	180.156		4.64(d)	0.26	0.08
Glycine	75.07	62.73	3.56(s)	5.53	
Glycerol	92.09382		3.55(dd, J = 6.5,11.7), 3.64(dd, J = 4.3,11.7), 3.77(m)		27.66
Isobutyric acid	88.11		1.05(d, J = 7.2)	0.02	0.08
Isovaleric acid	102.1317		0.90(d, J = 6.5), 1.95(m), 2.04(d, J = 7.4)	0.14	0.23
Lactic acid	90.08	20.23	4.12(q, J = 7.0), 1.33(d, J = 6.9)	0.42	0.87
Phenylalanine	165.19		7.33(d, J = 7.9), 7.38(t, J = 7.4), 7.43(t, J = 7.6)		0.04
L-Tartaric Acid	150.087		4.34(s)	0.03	0.05
Succinic Acid	118.09		2.40(s)	0.07	0.07
Sucrose	342.3		5.415(d, J = 3.9), 4.22(d,), 4.06(t,), 3.48(t,)	1.25	0.21
Valine	117.151		0.99(d, J = 7.0), 1.02(d, J = 7.0), 3.57(dt)	0.04	0.02
TSPA			−0.0159	0.186	0.186

^a^ ^1^H and ^13^C chemical shifts (in ppm) are referenced to 0.186 mM TSP at −0.0159 ppm [22]. ^b^ Determined from HSQC (in ppm). ^c^ Only chemical shifts of easily distinguished signals in the ^1^H-NMR spectra are reported. Chemical shifts that are not detectable and/or not distinguishable in either 1D or 2D NMR spectra are not provided.

**Table 2:** Amino acid sequence of several peptides in the fraction below 1 kDa, identified by tandem mass spectrometry.

№	Amino Acid Sequence of Peptides	Experimental *m*/*z* (Da)	[M+H]+ (Da)	Calculated Monoisotopic Mass (Da)	pI
P1	LGHDVH	339.165 [M+2H]^2+^	677.328	676.33	5.97
P2	LFSNQLFN	491.752 [M+2H]^2+^	982.504	981.49	5.52
P3	DQDSHPYSGP	551.725 [M+2H]^2+^	1102.450	1101.44	4.20
P4	LGLGNGGAGGGLVGG	578.306 [M+H]^2+^	1155.612	1154.60	5.52
P5	NNTVCGV	706.322 [M+2H]^+^	706.322	705.31	5.52
P6	LLMGPEV	379.710 [M+2H]^2+^	758.412	757.40	4.00
P7	AAGLAGAGNGGG	436.712 [M+2H]^2+^	872.424	871.41	5.57
P8	ACAATCGVEDGV	1095.539 [M+H]^+^	1095.539	1094.44	3.67

## Supplementary Materials

The following are available online at https://www.mdpi.com/2218-1989/10/9/360/s1, Figure S1: Typical 2D JRES spectrum of lyophilized slime from *Helix aspersa* (<1 kD) in the range from 5.0 to 9.0 ppm, pH = 7.35, 298.0 K, Figure S2: Typical 2D JRES spectrum of lyophilized slime from *Helix aspersa* (<1 kD) in the range from 4.5 to 0.5 ppm, pH = 7.35, 298.0 K, Figure S3: Typical 1D selective TOCSY with water suppression of lyophilized slime from *Helix aspersa* (<3 kD) revealing the metabolite lactic acid (lactate), Figure S4: Superposition of ^1^H-NMR spectra of lyophilized slime from *Helix aspersa* (<3 kD) (A) freshly prepared and (B) after 24 h at room temperature. There are no visible changes in the spectra, Figure S5: ^1^H,^13^C HSQC spectrum of lyophilized slime from *Helix aspersa* (<3 kD) (pH 7.35, 298.0 K). The spectral regions devoid of signals have been cut away, Figure S6: Typical ^1^H,^1^H COSY NMR spectrum of lyophilized slime from *Helix aspersa* (<3 kD) (pH 7.35, 298.0 K). The expansion of the 4.5–0.5 ppm region is shown. Figure S7: Typical ^1^H NMR spectrum of a buffer sample prepared under identical conditions but lacking snail mucus (pH 7.35, 298.0 K). The concentrations of contaminants (In this case: lactic acid at 1.32 ppm–0.05 mM; acetic acid at 1.91 ppm–0.03 mM; acetone at 2.22 ppm–0.03 mM and formic acid/formate at 8.44 ppm–0.03 mM) are usually at least an order of magnitude lower than the determined concentrations in the actual samples. **A**: 1D NOESY (noesygppr1d.2); **B**: 1D sequence (zgesgppe) with water suppression using excitation sculpting with gradients using perfect echo. Information S8: Information on the similarity of some identified peptides from *Helix aspersa* mucus (Table 2): (A) An alignment with known AMPs database by CAMPSing (http://www.campsign.bicnirrh.res.in/blast.php); (B) An alignment with protein data base by BLAST (https://blast.ncbi.nlm.nih.gov).
