# Supplementary material for: An 1H NMR- and MS-Based Study of Metabolites Profiling of Garden Snail Helix aspersa Mucus
Source: Metabolites. 2020 Sep 2;10(9):360. doi: 10.3390/metabo10090360 (PMC7570211; doi:10.3390/metabo10090360)
Supplement: Supplementary file 1 [file metabolites-10-00360-s001.pdf]

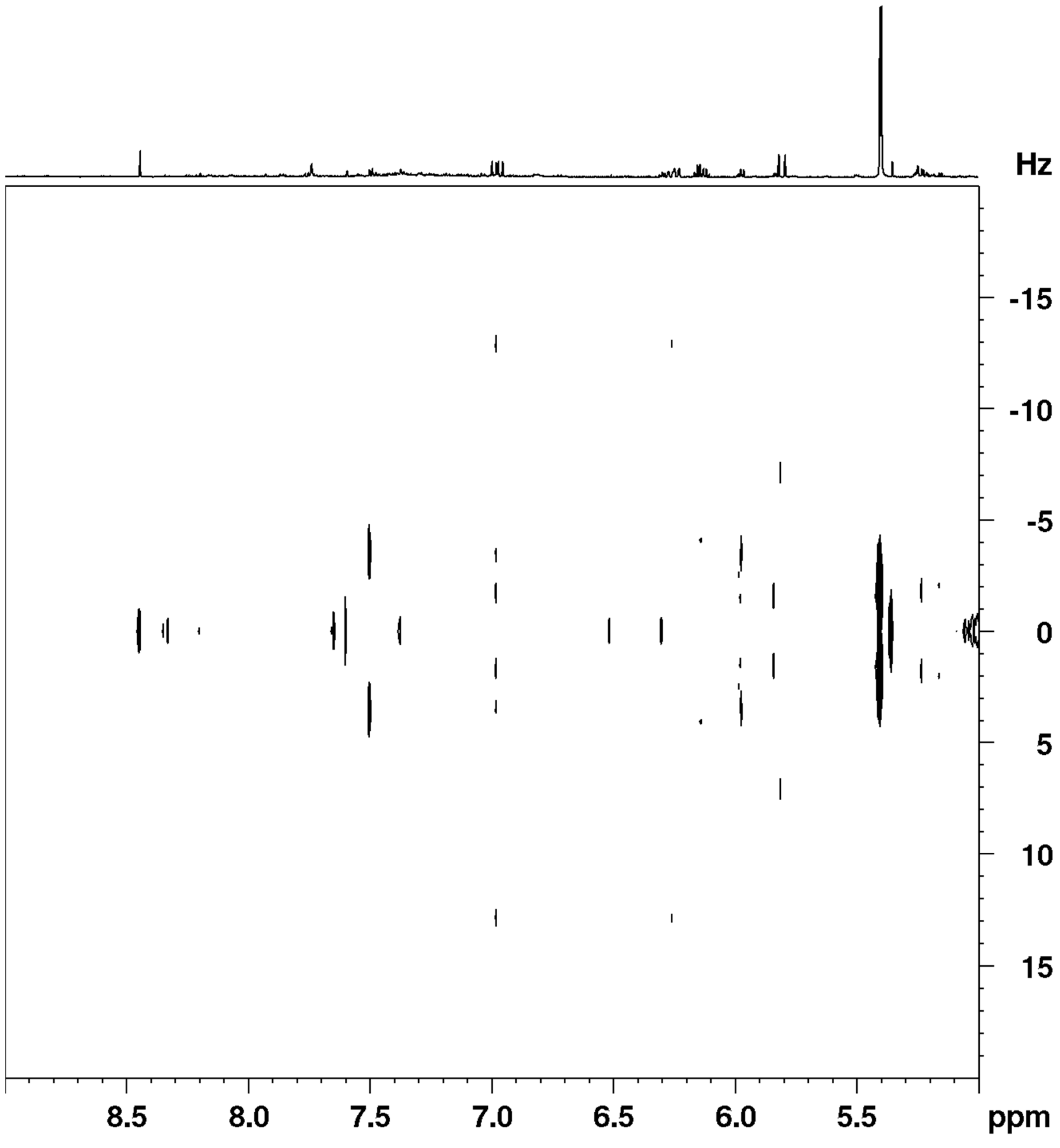

Figure S1. Typical 2D JRES spectrum of lyophilized slime from *Helix aspersa* (< 1 kD) in the range from 5.0 to 9.0 ppm, pH=7.35, 298.0 K.

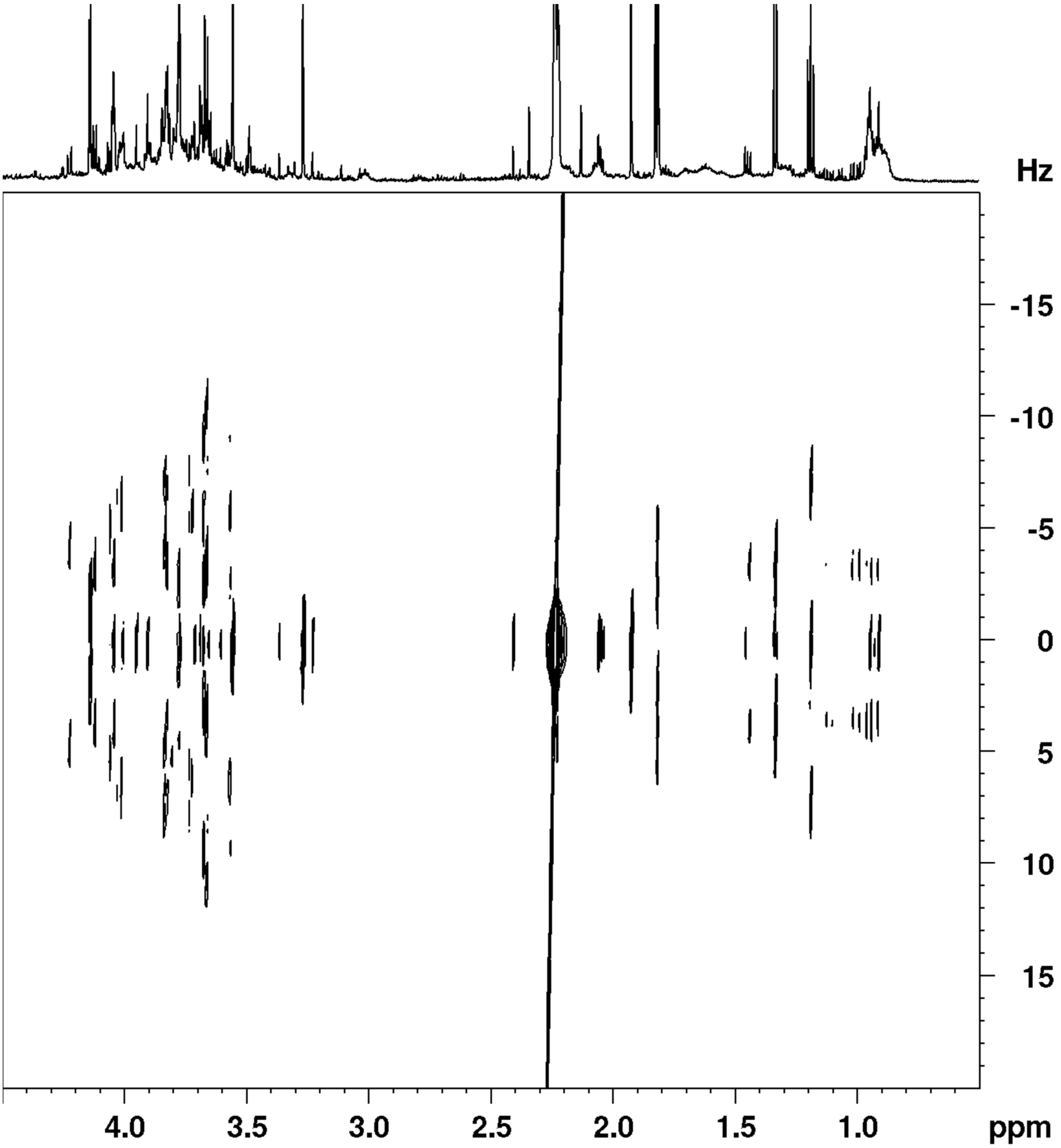

Figure S2. Typical 2D JRES spectrum of lyophilized slime from *Helix aspersa* (< 1 kD) in the range from 4.5 to 0.5 ppm, pH=7.35, 298.0 K.

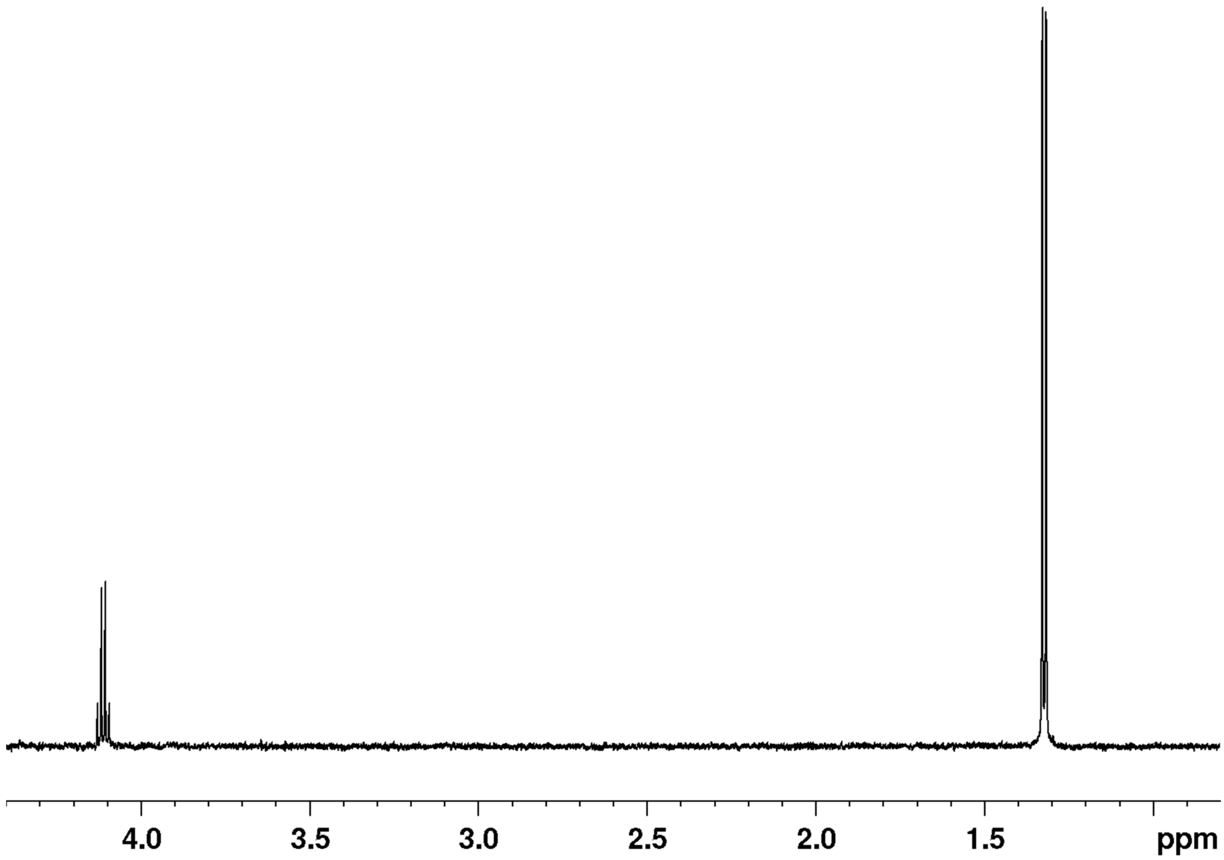

Figure S3. Typical 1D selective TOCSY with water suppression of lyophilized slime from *Helix aspersa* (< 3kD) revealing the metabolite lactic acid (lactate).

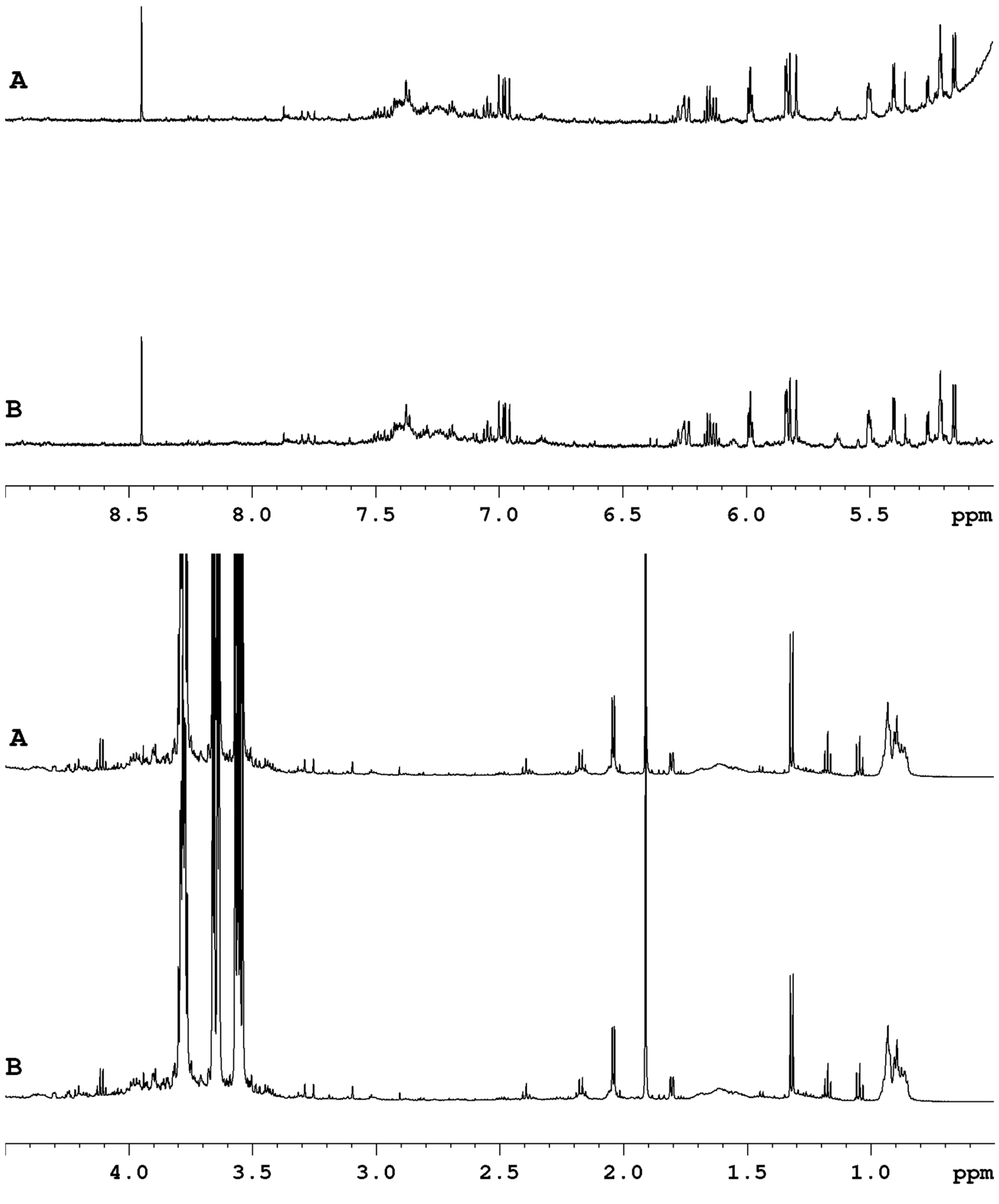

Figure S4. Superposition of ^{1}H-NMR spectra of lyophilized slime from *Helix aspersa* (< 3kD) **A)** freshly prepared and **B)** after 24 hours at room temperature. There are no visible changes in the spectra.

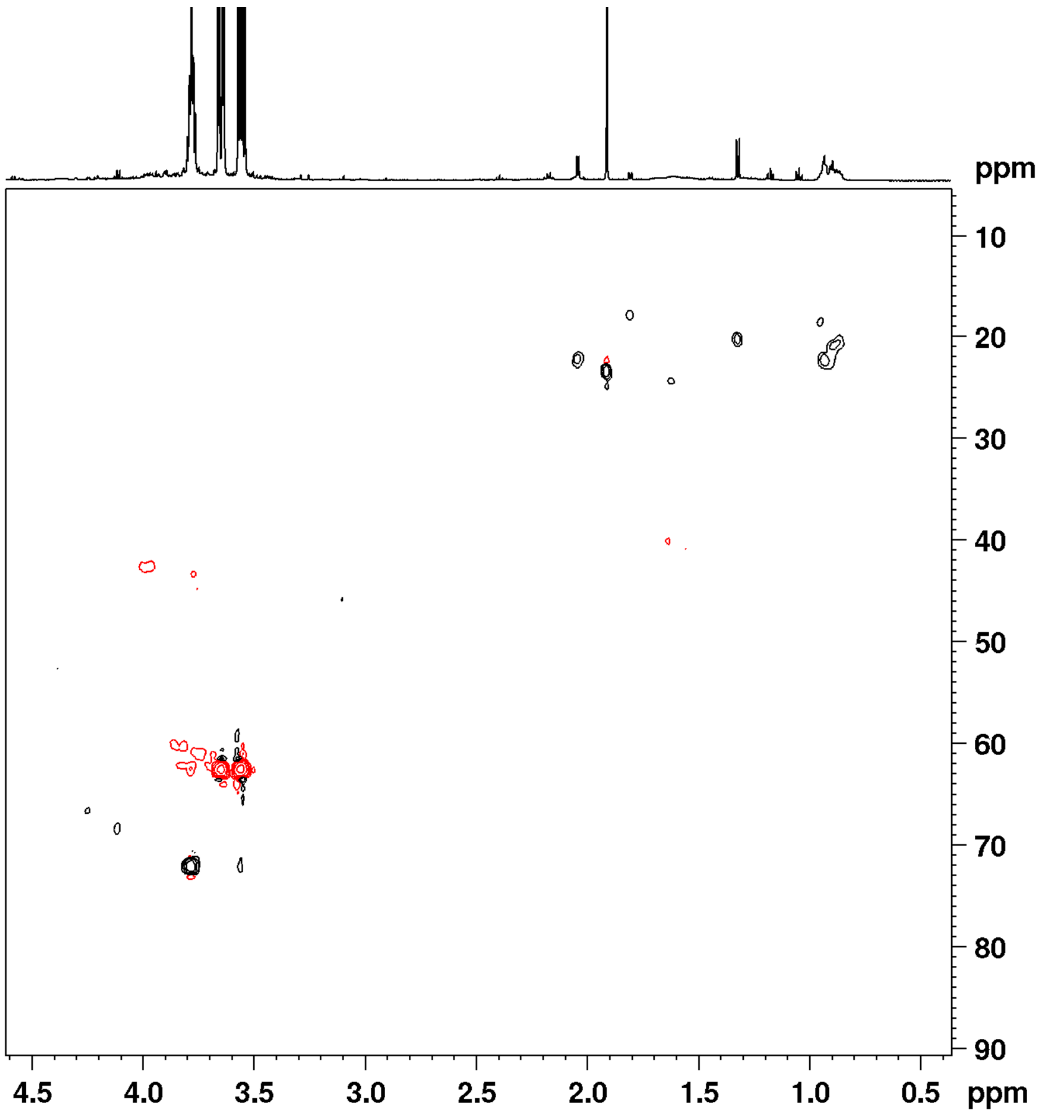

Figure S5. $^{1}H,^{13}C$ HSQC spectrum of lyophilized slime from *Helix aspersa* (< 3kD) (pH 7.35, 298.0 K). The spectral regions devoid of signals have been cut away.

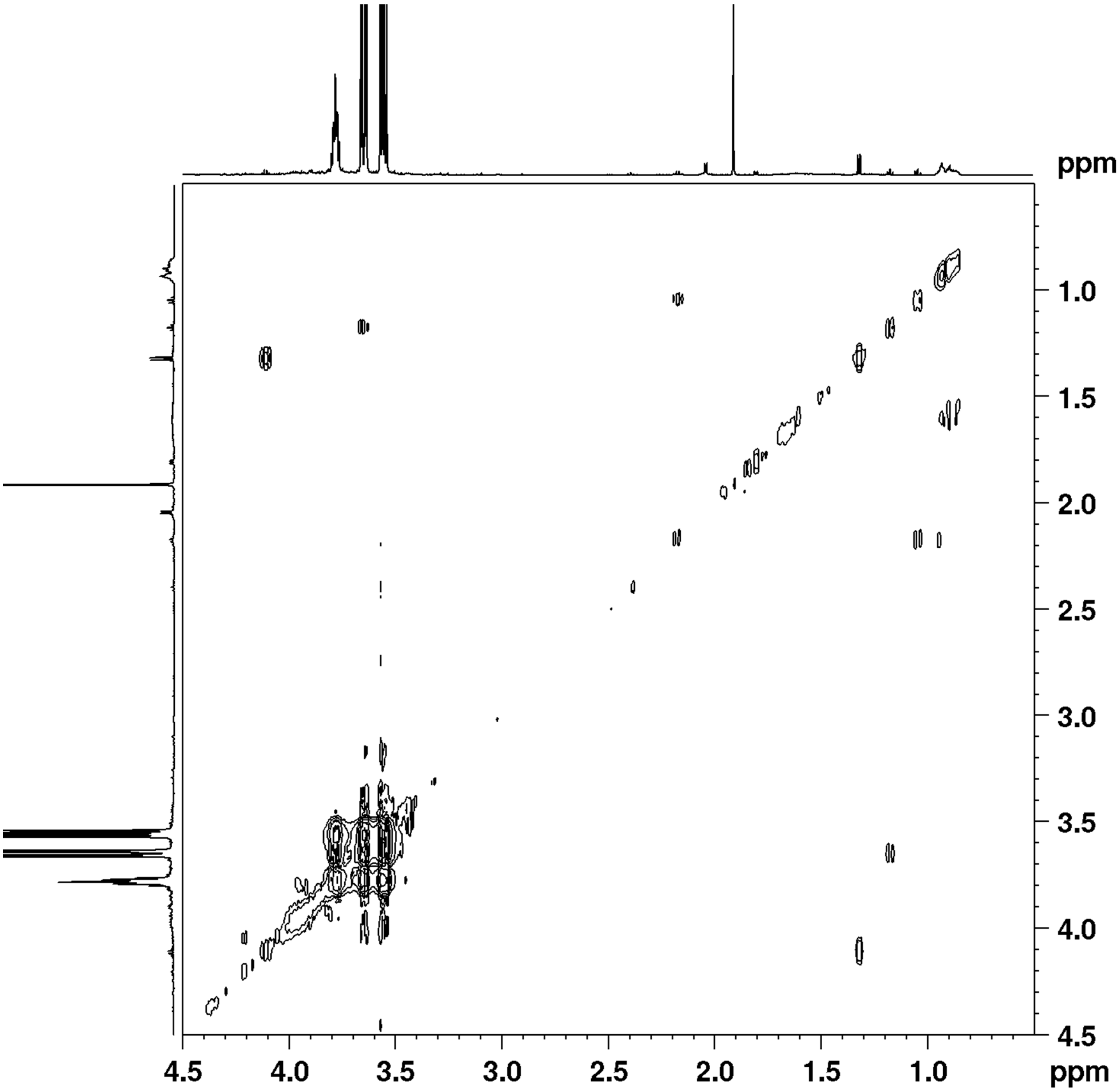

Figure S6. Typical ^{1}H,^{1}H COSY NMR spectrum of lyophilized slime from *Helix aspersa* (< 3kD) (pH 7.35, 298.0 K). The expansion of the 4.5-0.5 ppm region is shown.

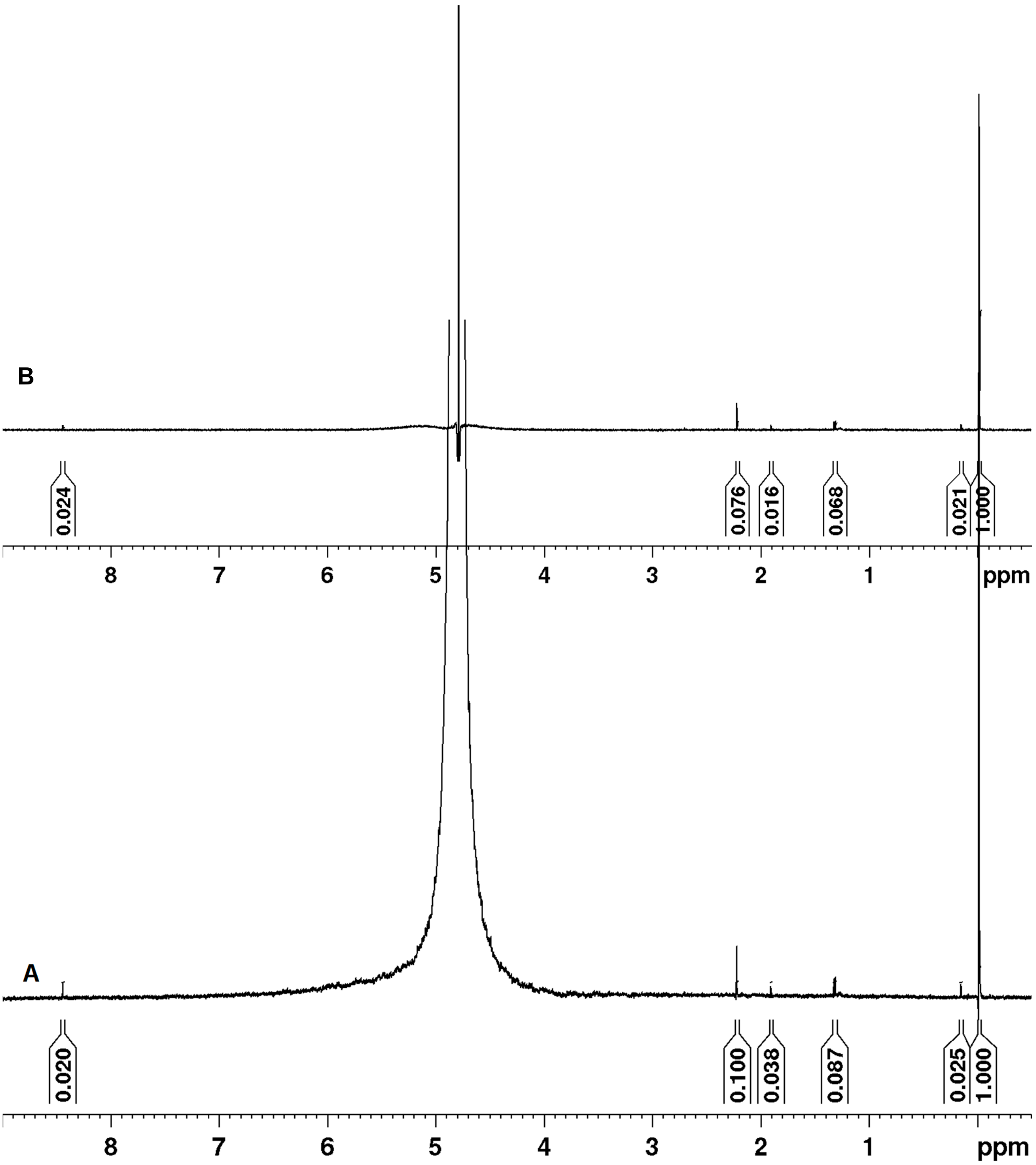

Figure S7. Typical ^{1}H NMR spectrum of a buffer sample prepared under identical conditions but lacking snail mucus (pH 7.35, 298.0 K). The concentrations of contaminants (In this case: lactic acid at 1.32 ppm – 0.05 mM; acetic acid at 1.91 ppm – 0.03 mM; acetone at 2.22 ppm – 0.03 mM and formic acid/formate at 8.44 ppm – 0.03 mM) are usually at least an order of magnitude lower than the determined concentrations in the actual samples. **A**: 1D NOESY (noesygppr1d.2); **B**: 1D sequence (zgesgppe) with water suppression using excitation sculpting with gradients using perfect echo.

Information S8: Information on the similarity of amino acid sequences of some identified peptides from *H. aspersa* mucus (Table 2):
A) An alignment with known AMPs database by CAMPSing
(http://www.campsign.bicnirrh.res.in/blast.php):

<u>Peptide P4: LGLGNGGAGGGLVGG</u>

> CAMPSQ3913| Ctenidin-1 (from *Cupiennius salei* [Wandering spider], UniProt P86798
Length 119, Identities = 11/15 (73%), Positives = 12/15 (80%), Gaps = 0/15 (0%)
Sequences producing significant alignment: Score = 28.0 bits (56), E -Value = 0.008

```
P4      1    LGLGNGGAGGGLVGG   15
             +GLG GG GGGL GG
Sbjct   61   VGLGGGGYGGGLGGG   75
```

Identities = 11/15 (73%), Positives = 11/15 (73%), Gaps = 1/15 (7%)
Sequences producing significant alignment: Score = 23.7 bits (46), E -Value = 0.23

```
P4      2    GLGNG-GAGGGLVGG   15
             GLG G G GGGL GG
Sbjct   71   GLGGGQGGGGGLGGG   85
```

> CAMPSQ3914| Ctenidin-3 (from Cupiennius salei [Wandering spider])
Length 120, Identities = 11/14 (79%), Positives = 11/14 (79%), Gaps = 0/14 (0%)
Sequences producing significant alignment: Score = 28.0 bits (56), E -Value = 0.008

```
P4      2    GLGNG-GAGGGLVGG   15
             GLG G G GGGL GG
Sbjct   71   GLGGGQGGGGGLGGG   85
```

Identities = 11/15 (73%), Positives = 11/15 (73%), Gaps = 1/15 (7%)
Sequences producing significant alignment: Score = 23.7 bits (46), E- Value = 0.23

```
P4      2    GLGNG-GAGGGLVGG   15
             GLG G G GGGL GG
Sbjct   83   GLGGGQGGGGGLGGG   97
```

<u>Peptide P7: AAGLAGAGNGGG</u>

> CAMPSQ5393| Cecropin TY1 (from *Tabanus yao* [Horsefly]), Antibacterial activity
Length=63, Identities = 8/10 (80%), Positives = 9/10 (90%), Gaps = 0/10 (0%)
Sequences producing significant alignment: Score = 21.5 bits (41), E - Value = 0.75

```
P7      1    AAGLAGAGNG   10
             AAG+AGA NG
Sbjct   54   AAGVAGALNG   63
```

B) An alignment with protein data base by BLAST (https://blast.ncbi.nlm.nih.gov):

Peptide P4: LGLGNGGAGGGLVGG

RecName: Full=Glycine-rich cell wall structural protein; Flags: Precursor [*Arabidopsis thaliana*]

Sequence ID: P27483.2, Length: 349, Number of Matches: 55

Range 1: 92 to 104 GenPeptGraphics Next Match Previous Match

Alignment statistics for match #1

Score	Expect	Identities	Positives	Gaps
28.2 bits(59)	0.74	12/14(86%)	12/14(85%)	1/14(7%)

```
P4      2     GLGNGGAGGGLVGG  15
              GLG GGAGGGL GG
Sbjct   92    GLG-GGAGGGLGGG  104
```

https://blast.ncbi.nlm.nih.gov/Blast.cgi?PROGRAM=blastp&PAGE_TYPE=BlastSearch&LINK_LOC=blasthome

RecName: putative protein Ll1785 [*Littorina littorea*]

Sequence ID: MBX96353.1, Length: 791, Number of Matches: 6

Range 1: 424 to 436 GenPeptGraphics Next Match Previous Match

Alignment statistics for match #1

Score	Expect	Identities	Positives	Gaps
30.3 bits(64)	0.90	11/13(85%)	11/13(84%)	0/13(0%)

```
P4      2     GLGNGGAGGGLVG  14
              GLG GGAGGGL G
Sbjct   424   GLGGGGAGGGLMG  436
```

Peptide P7: AAGLAGAGNGGG

RecName: Full=Voltage-dependent calcium channel gamma-8 subunit; AltName: Full=Neuronal voltage-gated calcium channel gamma-8 subunit; AltName: Full=Transmembrane AMPAR regulatory protein gamma-8; Short=TARP gamma-8 [Homo sapiens]

Sequence ID: Q8WXS5.3, Length: 425, Number of Matches: 5

Range 1: 319 to 330GenPeptGraphics Next MatchPrevious Match

Alignment statistics for match #1

Score	Expect	Identities	Positives	Gaps
30.3 bits(64)	0.075	11/12(92%)	11/12(91%)	0/12(0%)

```
P7      1     AAGLAGAGNGGG  12
              AAGLAGAG GGG
Sbjct   319   AAGLAGAGGGGG  330
```
